# Recurrent Strictly Unilateral Extra-Axial Intracranial Hemorrhage in Severe Hemophilia A Without Inhibitors Revealing a Suspected Dural Arteriovenous Fistula: A Case Report

**DOI:** 10.7759/cureus.106835

**Published:** 2026-04-11

**Authors:** Hind Zahiri, Ayad Ghanam, Hassnae Tkak, Manal Azizi, Maria Rkain

**Affiliations:** 1 Pediatric Medicine, Mohammed VI University Hospital, Faculty of Medicine and Pharmacy Mohammed 1st University, Oujda, MAR; 2 Pediatric Gastroenterology, CHU Mohammed VI Oujda, Oujda, MAR

**Keywords:** central nervous system bleeding, digital subtraction angiography(dsa), dural arteriovenous fistula (davf), extra-axial hematoma, factor viii deficiency, hemophilia-a, magnetic resonance angiography (mra), recurrent intracranial hemorrhage, subdural hematoma (sdh)

## Abstract

Intracranial hemorrhage is a feared complication of hemophilia A, and recurrence may prompt evaluation beyond systemic hemostatic factors. We report an 18-year-old male with severe hemophilia A and no current inhibitors who experienced three episodes of spontaneous, strictly right-sided extra-axial intracranial hemorrhage (epidural and subdural) over four years. Each event presented with headache and vomiting without antecedent trauma and was managed conservatively with high-dose factor VIII replacement and close neurologic monitoring, resulting in complete clinical recovery and radiologic resolution. After the third ipsilateral event, repeated inhibitor testing remained negative, and systemic risk factor evaluation was unrevealing. Brain magnetic resonance angiography (MRA) demonstrated an ectatic right parietal cortical venous structure draining into the superior sagittal sinus with suspected arteriovenous shunting, raising concern for a dural arteriovenous fistula (DAVF). Digital subtraction angiography (DSA) was recommended for definitive diagnosis, but was not immediately available, and the patient is awaiting transfer. This case highlights that recurrent strictly unilateral extra-axial hemorrhage in hemophilia should prompt investigation for a focal structural or vascular etiology such as DAVF, particularly in resource-limited settings.

## Introduction

Hemophilia A is an inherited bleeding disorder transmitted through an X-linked recessive pattern. Its clinical severity depends on the amount of functional clotting factor VIII present in the blood [1]. While bleeding into joints and muscles is characteristic of hemophilia, intracranial hemorrhage (ICH) represents its most serious complication. These brain bleeds can be life-threatening and are often linked to significant long-term neurological impairments in those who survive [2,3]. A recent systematic review and meta-analysis demonstrated that the pooled incidence of ICH in persons with hemophilia is significantly higher than in the general population, with 35% to 58% of these events classified as strictly spontaneous [3].

Several factors have been associated with ICH risk in hemophilia, including disease severity, age-related vulnerability (particularly early childhood and later adulthood), prior ICH, and inhibitors, as well as comorbidities and care-related determinants. In contemporary real-world data from the American Thrombosis and Hemostasis Network (ATHN) dataset, identified risk factors for ICH in persons with hemophilia A included age 2-12 years, human immunodeficiency virus (HIV), hypertension, and never having received factor treatment or prophylaxis [4]. When ICH is recurrent, especially with a strictly unilateral distribution, evaluation should extend beyond systemic hemostatic factors to consider a local structural or vascular etiology. Recent cohort studies of hemophilia-associated ICH have mainly focused on systemic and treatment-related risk factors rather than the prevalence of structural cerebrovascular lesions [4,5]. Therefore, recurrent strictly unilateral hemorrhage is clinically unusual in hemophilia and should prompt targeted vascular imaging for a focal lesion such as a dural arteriovenous fistula (DAVF) [5].

DAVFs are rare intracranial vascular shunts that may present with hemorrhage or focal neurologic deficits. Digital subtraction angiography (DSA) remains the diagnostic reference standard, while magnetic resonance imaging (MRI)/magnetic resonance angiography (MRA) can provide a noninvasive first-line assessment [6].

While most literature has focused on systemic risk factors [4], structural cerebrovascular abnormalities as contributors to recurrent, localized bleeding events in hemophilia remain poorly characterized. Here, we describe a unique case of recurrent unilateral extra-axial hemorrhage in a young adult with severe hemophilia A and no detectable inhibitors, raising suspicion for an underlying DAVF and expanding the differential for recurrent ICH in this population.

## Case presentation

An 18-year-old male with severe hemophilia A (baseline factor VIII activity: 0.6%) was followed in our center. The diagnosis was established at the age of six months following post-circumcision bleeding, with no known family history of bleeding disorders. Initial management consisted of fresh-frozen plasma administration during bleeding episodes because factor VIII concentrates were unavailable. At the age of nine years, on-demand treatment with plasma-derived factor VIII (pdFVIII, Octanate® (Octapharma AG, Lachen, Switzerland) was initiated. A target joint was identified at the level of the right ankle.

**Figure 1:**
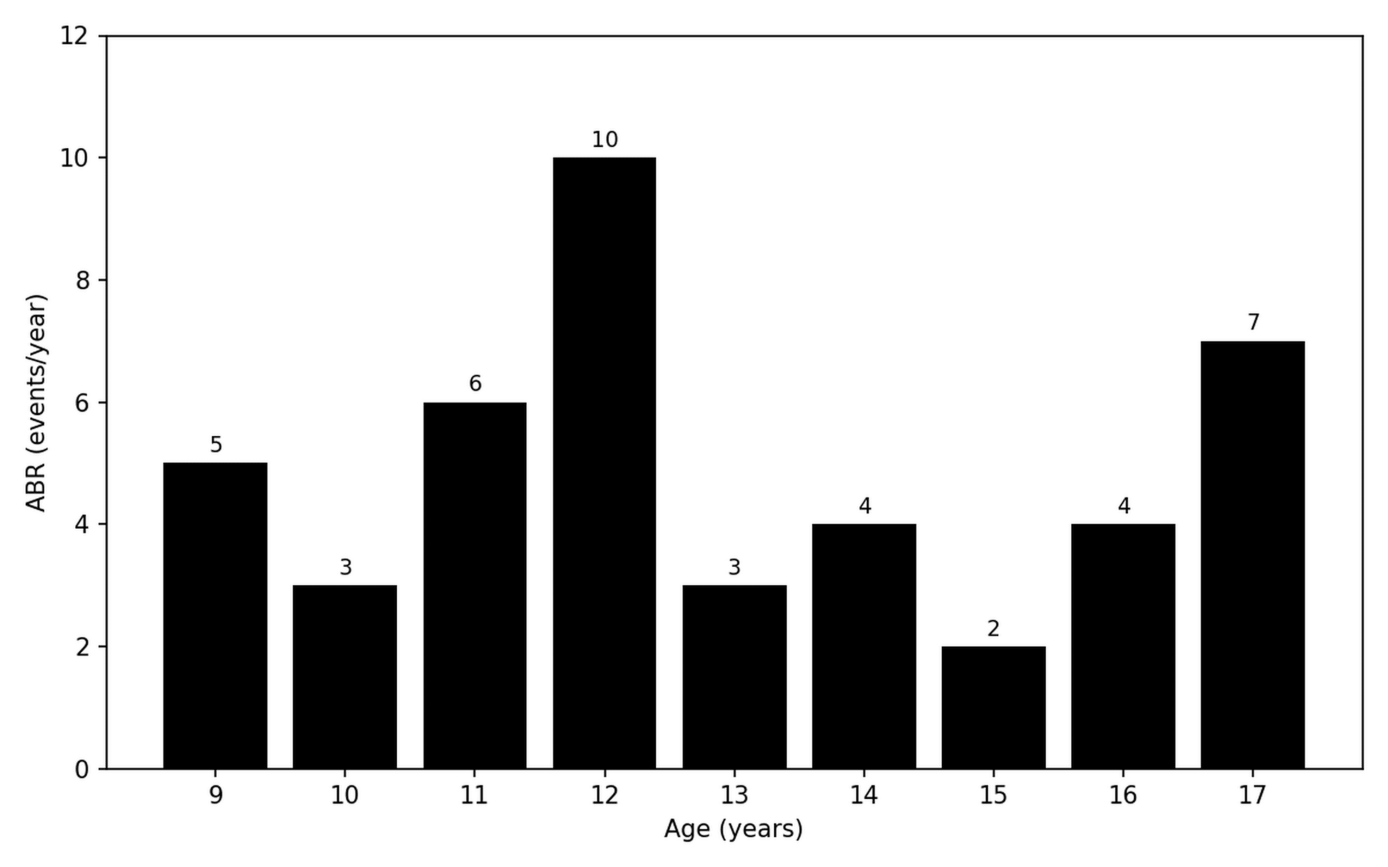
Annualized bleeding rate (ABR) by age (9–17 years) in a patient with severe hemophilia A. The rise in ABR coincided with the development of a low-titer factor VIII inhibitor. After initiation of immune tolerance induction (ITI), inhibitor testing became negative and ABR decreased, followed by sustained lower bleeding rates under continued factor VIII prophylaxis.

At 11 years of age, due to an increased frequency of hospitalizations for bleeding episodes with an annualized bleeding rate (ABR) of 10, inhibitor screening revealed a low-titer inhibitor (3 BU/mL, Bethesda/Nijmegen method), consistent with a low-responder profile. Immune tolerance induction (ITI) was initiated at the age of 12 years using factor VIII (20 IU/kg twice weekly), resulting in inhibitor eradication after nine months. Prophylaxis with factor VIII was subsequently maintained using the same regimen, leading to a reduction in hospitalization frequency (Figure 1).

The patient experienced three episodes of spontaneous extra-axial intracranial hemorrhage, all involving the right hemisphere (Table 1).

**Table 1:** Summary of the three spontaneous, strictly right-sided episodes of extra-axial intracranial hemorrhage in a patient with severe hemophilia A. CT: computed tomography; DSA: digital subtraction angiography; FVIII: factor VIII; MRA: magnetic resonance angiography; DAVF: dural arteriovenous fistula.

Episode	Age	Clinical presentation	Imaging findings	Treatment	Outcome
1	14 years	Headache and nausea for over 48 hours; no trauma	CT: right fronto-parietal and temporal epidural hematoma; no significant midline shift	High-dose FVIII replacement with activity monitoring; close neurological monitoring	Complete clinical recovery; complete radiologic resolution at 1 month
2	17 years 3 months	Headache and vomiting, no trauma; prophylaxis had been interrupted due to FVIII shortage	CT: right fronto-temporal subdural hematomas associated with a right frontal epidural hematoma; cerebral edema; 5 mm midline shift to the left	High-dose FVIII replacement with activity monitoring; dexamethasone; close observation	Complete clinical recovery; complete radiologic resolution at 1 month
3	18 years 1 month	Headache and vomiting over 24 hours; left upper-limb paresthesia and mild weakness; no trauma	CT: right frontotemporoparietal subdural hematoma with cerebral edema and associated meningeal hemorrhage along the falx cerebri and tentorium cerebelli; 5 mm midline shift to the left. MRA: ectatic right parietal cortical venous structure draining into the superior sagittal sinus with suspected arteriovenous shunting, concerning for DAVF	High-dose FVIII replacement with activity monitoring; dexamethasone; close neurologic monitoring; referral for DSA	Progressive neurologic improvement; complete deficit resolution within 3 days; awaiting DSA

**Figure 2:**
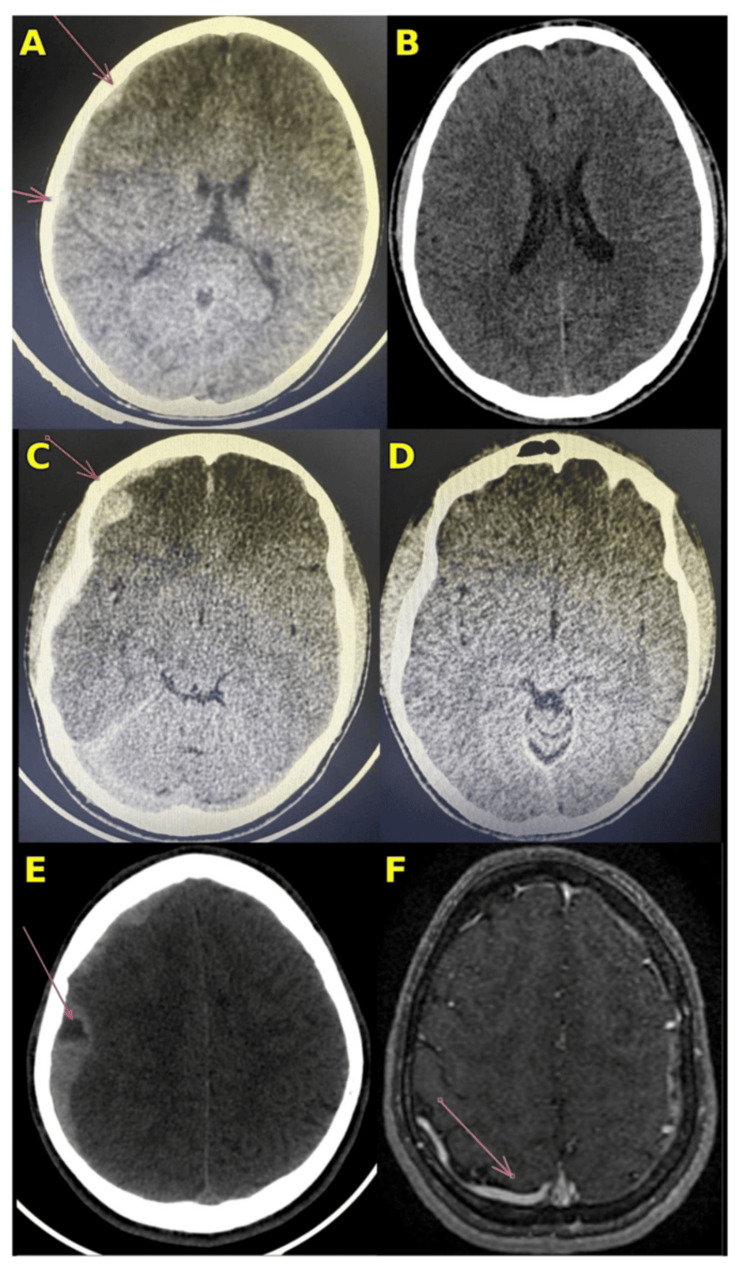
Neuroimaging findings across three episodes of recurrent strictly right-sided extra-axial intracranial hemorrhage and MRI/MRA features suggestive of a dural arteriovenous fistula. (A) Axial non-contrast brain CT at age 14 showing a right frontoparietotemporal epidural hematoma (orange and pink arrows) without significant midline shift. (B) Follow-up axial non-contrast CT one month later demonstrating complete radiologic resolution. (C) Axial non-contrast CT at age 17 years and 3 months showing a right frontotemporal subdural hematoma associated with a right frontal epidural hematoma (orange arrow) and mass effect with an approximately 5-mm leftward midline shift. (D) Follow-up axial non-contrast CT one month later demonstrating complete resolution. (E) Axial non-contrast CT at age 18 years and 1 month showing a heterogeneous right frontotemporoparietal subdural hematoma (pink arrow) with and an approximately 5-mm leftward midline shift. (F) Post-contrast axial T1-weighted MR angiography demonstrating an ectatic right parietal cortical venous structure (pink arrow) draining into the superior sagittal sinus, raising suspicion for arteriovenous shunting and a dural arteriovenous fistula.

The first episode occurred at the age of 14 years. The patient presented with headache and nausea evolving over 48 hours, without fever, trauma, nonsteroidal anti-inflammatory drug (NSAID) use, or other associated bleeding, and with reported good adherence to prophylaxis. In the context of suspected intracranial hemorrhage, factor VIII (20 IU/kg) was administered prior to imaging. Brain computed tomography (CT) revealed a right fronto-parietal and temporal biconvex hyperdense lesion (maximum thickness 5 mm in the frontal region), consistent with spontaneous epidural hematomas, without significant midline shift (Figure 2A). Treatment consisted of high-dose factor VIII (100 IU/kg every 8 hours) with monitoring of factor VIII activity, targeting 100% for 48 hours, followed by dose adjustment to maintain levels above 80% during the first week and above 50% during the second and third weeks. Neurosurgical evaluation favored conservative management with close neurological monitoring. Inhibitor testing at that time was negative (0 BU/mL). Clinical evolution was favorable, and follow-up CT at one month showed complete resolution (Figure 2B). Repeat inhibitor testing at six months remained negative.

The second episode occurred at the age of 17 years and three months. Notably, the patient had not received prophylaxis during the preceding year due to a shortage of factor VIII concentrate. He presented with headache and vomiting without preceding trauma. CT imaging demonstrated right fronto-temporal subdural hematomas (up to 4 mm thick), associated with a right frontal epidural hematoma (maximum thickness 13 mm). Cerebral edema was suggested by sulcal effacement, with a midline shift of 5 mm toward the left (Figure 2C). Management consisted of high-dose factor VIII replacement therapy with activity monitoring, in addition to dexamethasone (0.25 mg/kg every 6 hours) administered for cerebral edema. Neurosurgical assessment again supported conservative management with close observation. Inhibitor testing remained negative. Follow-up CT at one month demonstrated complete resolution (Figure 2D). Prophylaxis with factor VIII (20 IU/kg twice weekly) was resumed, and inhibitor testing at six months remained negative.

The current episode occurred at the age of 18 years and one month, approximately 10 months after the previous event. The patient presented with headache and vomiting evolving over 24 hours, occurring three days after his last prophylactic dose. There was no history of trauma or NSAID use. He also reported paresthesia of the left upper limb. On admission, he was hemodynamically stable and in no respiratory distress (blood pressure 110/57 mmHg, heart rate 110 bpm, respiratory rate 17 breaths/min, oxygen saturation 97%, temperature 37.2°C, weight 55 kg). Neurological examination revealed no impairment of consciousness (Glasgow Coma Scale 15/15), with reactive pupils and no seizures. A mild motor deficit of the left upper limb was noted, with muscle strength graded at 4/5. Fundoscopic examination was normal. Brain CT revealed a right frontotemporoparietal subdural hematoma with heterogeneous density (maximum thickness 17 mm), associated with cerebral edema and spontaneous hyperdensity of the falx cerebri and tentorium cerebelli, consistent with associated meningeal hemorrhage. A midline shift of 5 mm toward the left was noted, without skull fracture (Figure 2E).

Factor VIII replacement therapy was immediately initiated according to central nervous system (CNS) bleeding protocols, similar to previous episodes, with activity monitoring. Dexamethasone (0.25 mg/kg every 6 hours) was administered for cerebral edema. Neurosurgical consultation again concluded that conservative management was appropriate given the absence of neurological deterioration. The neurological symptoms showed progressive improvement after the initiation of treatment, with complete resolution of the deficit within three days.

Given this third strictly right-sided intracranial hemorrhage, inhibitor recurrence was suspected because of repeated exposure to high doses of factor VIII. However, inhibitor testing remained negative (0 BU/mL). Systemic evaluation was unremarkable, including a normal platelet count (246,000/µL; reference range: 150,000-450,000/µL), normal 24-hour ambulatory blood pressure monitoring, and negative serology for HIV, hepatitis B, and hepatitis C.

The strictly unilateral recurrence prompted investigation for a local vascular cause. Cerebral magnetic resonance angiography (MRA) revealed an ectatic cortical vascular structure in the right parietal region draining into the superior sagittal sinus, with suspected arteriovenous shunting arising from branches of the right internal carotid artery. MR venography (MRV) was not obtained because it was not available at the time of evaluation in our setting. These findings were highly suggestive of a dural arteriovenous fistula (DAVF) (Figure 2F). Confirmation by digital subtraction angiography (DSA) was indicated; however, due to its unavailability in our setting, the patient is currently awaiting transfer for further evaluation.

## Discussion

Hemophilia A is an X-linked inherited bleeding disorder caused by factor VIII (FVIII) deficiency, leading to impaired thrombin generation and a tendency to spontaneous bleeding (especially into joints and muscles), and life-threatening hemorrhage, such as intracranial bleeding, in severe cases. Disease severity is commonly classified by baseline FVIII activity (severe <1 IU/dL, moderate 1-5 IU/dL, mild 5-<40 IU/dL) [1]. Modern management is centered on hemostatic therapy with FVIII concentrates (plasma-derived or recombinant), used either on-demand to treat bleeds or preferably as regular prophylaxis, which is considered the standard of care for severe hemophilia [7]. Prophylaxis in hemophilia A can be delivered using factor VIII concentrates with different intensities (high, intermediate, or low-dose regimens), typically varying by dose and infusion frequency (e.g., 25-40 IU/kg every other day or three times weekly for high-dose vs 10-15 IU/kg two to three times weekly for low-dose schedules) [1]. In our center, due to modest FVIII availability, our patient received a low-intensity FVIII prophylaxis regimen (20 IU/kg twice weekly).

A major complication is the development of FVIII inhibitors, which can render FVIII replacement ineffective. Immune tolerance induction (ITI) is an established approach to eradicate inhibitors [8]. In our patient, a low-titer FVIII inhibitor (3 BU/mL; Nijmegen-modified Bethesda assay) [9] was detected at 11 years of age, consistent with a low-responder profile. ITI was initiated at 12 years using a low-intensity FVIII regimen (20 IU/kg twice weekly) and achieved inhibitor eradication after nine months.

In addition, non-factor prophylaxis has expanded options. For instance, emicizumab, a bispecific monoclonal antibody that mimics FVIIIa function, is recommended as prophylaxis in hemophilia A with or without inhibitors in contemporary guidance, offering subcutaneous administration and reduced treatment burden [10].

Although central nervous system (CNS) bleeding represents a minority of bleeding episodes in hemophilia (estimated at <5% by the World Federation of Hemophilia (WFH)), it is among the most life-threatening events and requires emergency management [1]. A large systematic review and meta-analysis (45 studies; >54,000 patients) reported that, across all ages, pooled ICH incidence and mortality were 2.3 and 0.8 per 1,000 person-years, respectively; rates were higher in children and young adults (7.4 and 0.5 per 1,000 person-years), and neonatal cumulative incidence was 2.1% per 100 live births. Importantly, ICH was described as spontaneous in 35%-58% of cases [3]. 

ICH in hemophilia is a medical emergency and requires immediate hemostatic correction. Current guidance recommends administering FVIII promptly when CNS (central nervous system) bleeding is suspected, ideally before neuroimaging, followed by urgent Computed Tomography/MRI, close neurologic monitoring, and early neurosurgical consultation; if ICH is confirmed, FVIII levels should be maintained at hemostatic targets for an adequate duration (often 10-14 days) with laboratory monitoring [1].

For hemophilia A without inhibitors, recommended practice patterns target high FVIII levels early. In the WFH practice-pattern table for intracranial hemorrhage, a higher-dose approach targets peak FVIII 80-100 IU/dL initially (for 1-7 days) followed by maintenance around 50 IU/dL (for 8-21 days), while lower-dose practice patterns list initial peaks of 50-80 IU/dL (for 1-3 days) with subsequent maintenance targets of 20-40 IU/dL (for 8-14 days) [1].

The choice between conservative and surgical management of extra-axial hematomas is individualized and largely driven by neurological status and radiologic mass effect; when surgery is indicated, perioperative planning must ensure sufficient FVIII coverage (or bypassing agents if inhibitors are present) [1,11]. In our patient, all three spontaneous extra-axial hemorrhages were managed conservatively because consciousness was preserved and no progressive neurological deterioration occurred, with rapid clinical improvement under high-dose FVIII replacement and complete radiologic resolution on follow-up imaging.

Adjunctive corticosteroids are not routinely recommended for spontaneous intracerebral hemorrhage and should not be used prophylactically to treat elevated intracranial pressure [12]. In addition, randomized trials in chronic subdural hematoma reported worse functional outcomes and higher complication rates with dexamethasone, arguing against routine steroid therapy [13]. In our patient, dexamethasone was used only as an adjunct for radiologic edema with mass effect under neurosurgical guidance.

Prophylactic antiseizure medication is not recommended in the absence of clinical or electrographic seizures; antiseizure drugs should be given for documented (or strongly suspected) seizures, with EEG monitoring when indicated [12]. Accordingly, in our patient, prophylactic antiseizure therapy was not routinely used in the absence of documented seizures, and management relied on close neurologic monitoring with escalation if seizures were to occur.

Risk factors for ICH in hemophilia are multifactorial and include both hemophilia-related and general vascular/comorbidity determinants. In a contemporary real-world analysis of the U.S. ATHN dataset (published in 2024), characteristics associated with higher ICH risk included age 2-12 years, HIV, hepatitis C, hypertension, and never having received FVIII treatment or prophylaxis; prophylaxis showed a protective association, while inhibitor status was not statistically associated with ICH in that analysis [4]. The strictly ipsilateral recurrence of three spontaneous extra-axial intracranial hemorrhages in our patient (all confined to the right hemisphere) prompted us to broaden the etiologic work-up beyond systemic hemophilia-related determinants. Repeated inhibitor testing remained negative, ambulatory blood pressure monitoring was normal, and HIV/HBV/HCV serologies were negative, making common systemic contributors less likely and strengthening the suspicion of a focal structural or vascular source of bleeding.

Vascular malformations are well-recognized causes of ICH in younger individuals, particularly brain arteriovenous malformations (bAVMs) and dural arteriovenous fistulas (DAVFs) [14,5]. DAVFs are abnormal arteriovenous shunts within the dura, and their clinical course is largely driven by venous drainage patterns; lesions with retrograde venous drainage and cortical venous reflux are considered aggressive and carry an increased risk of hemorrhage and neurological morbidity [5]. Digital subtraction angiography (DSA) remains the diagnostic gold standard to define arterial feeders, venous outflow, and cortical reflux, and to guide definitive therapy, while MRI/MRA may serve as a noninvasive first-line test that raises suspicion and directs further evaluation [15].

Although intraparenchymal hemorrhage is the most common hemorrhagic presentation, isolated subdural hematoma due to DAVF has been reported and remains a relevant diagnostic consideration in patients with spontaneous extra-axial hemorrhage. The proposed mechanism is rupture of arterialized, cortical, leptomeningeal, or bridging venous structures exposed to abnormal venous pressure. However, this remains poorly characterized compared to the mechanisms of parenchymal hemorrhage. [16]. In our case, despite the lack of immediate DSA, the combination of a strictly unilateral recurrence and signs of arteriovenous shunting on the MRA was sufficient to broaden the etiologic work-up and expedite referral for angiographic confirmation, illustrating a pragmatic diagnostic pathway in resource-limited contexts.

Recent reviews characterize intracranial DAVFs as predominantly acquired and most often associated with dural sinus thrombosis, venous hypertension, trauma, or prior craniotomy. They do not identify hemophilia as a recognized predisposing factor for DAVF formation. Accordingly, published evidence supporting hemophilia as a trigger for DAVF formation (e.g., via bleeding-driven angiogenesis) remains limited and unproven [5,17]. In our patient with severe hemophilia A and recurrent strictly right-sided extra-axial hemorrhage, a previously silent DAVF could plausibly have been unmasked, as DAVF hemorrhagic behavior is largely determined by venous drainage patterns, particularly cortical venous drainage reflux.

If a DAVF is confirmed, definitive management is typically endovascular (often requiring catheterization under general anesthesia), and many DAVF embolization series describe the procedure being performed with systemic heparinization, which adds an extra hemostatic challenge in a patient with an inherited bleeding disorder [18]. In hemophilia A, any invasive neurointerventional strategy therefore requires multidisciplinary peri-procedural planning (hematology-anesthesia-neurointervention) and factor coverage at major surgery targets (practice patterns often aiming for peak FVIII 80-100 IU/dL initially), with careful post-procedure monitoring and duration of support tailored to bleeding risk [19].

## Conclusions

This case illustrates an exceptional pattern of intracranial bleeding in severe hemophilia A, where three episodes of spontaneous, strictly right-sided extra-axial hemorrhage occurred in the absence of current inhibitors and resolved with optimized factor VIII replacement. The consistent ipsilateral recurrence was the key clinical signal that broadened the differential beyond systemic hemostatic vulnerability and led to targeted vascular assessment. In a resource-limited setting where immediate digital subtraction angiography is not available, MRI/MRA served as a pivotal non-invasive bridge by revealing findings suggestive of a possible dural arteriovenous fistula, rather than confirming its presence, and thereby enabling timely referral for definitive angiographic evaluation and further management. Recognizing atypical bleeding topography and integrating early hematology, neurosurgery, and neurointervention coordination may help prevent diagnostic delay and improve outcomes in similarly complex presentations.

## References

[1] Srivastava A, Santagostino E, Dougall A (2020). WFH Guidelines for the Management of Hemophilia, 3rd edition. Haemophilia.

[2] Andersson NG, Wu R, Carcao M (2020). Long-term follow-up of neonatal intracranial haemorrhage in children with severe haemophilia. Br J Haematol.

[3] Zwagemaker AF, Gouw SC, Jansen JS (2021). Incidence and mortality rates of intracranial hemorrhage in hemophilia: a systematic review and meta-analysis. Blood.

[4] Hu J, Chandler M, Manuel CM (2024). Risk of intracranial hemorrhage in persons with hemophilia A in the United States: real-world retrospective cohort study using the ATHNdataset. J Blood Med.

[5] Maciejewski K, Pinkiewicz M, Mruk B, Knap D, Zaczyński A, Walecki J, Zawadzki M (2025). A practical approach to intracranial dural arteriovenous fistulas: pathogenesis, classification and management. J Clin Med.

[6] Chen X, Ge L, Wan H, Huang L, Jiang Y, Lu G, Zhang X (2022). Overview of multimodal MRI of intracranial Dural arteriovenous fistulas. J Interv Med.

[7] Rezende SM, Neumann I, Angchaisuksiri P (2024). International Society on Thrombosis and Haemostasis clinical practice guideline for treatment of congenital hemophilia A and B based on the Grading of Recommendations Assessment, Development, and Evaluation methodology. J Thromb Haemost.

[8] Tiede A, Alberio L (2020). The art of detecting antibodies against factor VIII. Hamostaseologie.

[9] Reding MT (2025). New therapies in hemophilia: extend the half-life, mimic, or rebalance?. Hematology Am Soc Hematol Educ Program.

[10] Alcedo Andrade PE, Mannucci PM, Kessler CM (2024). Emicizumab: the hemophilia A game-changer. Haematologica.

[11] Ninan MK, Chennupati PK, Gurrala SR (2025). Intracranial hemorrhage in a patient of hemophilia A with factor VIII inhibitors positive: a case report. J Neuroanaesthesiol Crit Care.

[12] Greenberg SM, Ziai WC, Cordonnier C (2022). 2022 Guideline for the Management of Patients With Spontaneous Intracerebral Hemorrhage: A Guideline From the American Heart Association/American Stroke Association. Stroke.

[13] Hutchinson PJ, Edlmann E, Bulters D (2020). Trial of dexamethasone for chronic subdural hematoma. N Engl J Med.

[14] Kim H, Nelson J, McCulloch CE (2025). Risk of future hemorrhage from unruptured brain arteriovenous malformations: the Multicenter Arteriovenous Malformation Research Study (MARS). JAMA Neurol.

[15] Shekar V, Lucke-Wold B (2025). Evolving management paradigms in dural arteriovenous fistulas: from classification to personalized endovascular therapy. Biomedicines.

[16] Li G, Zhang Y, Zhao J, Zhu X, Yu J, Hou K (2019). Isolated subdural hematoma secondary to Dural arteriovenous fistula: a case report and literature review. BMC Neurol.

[17] Sammoud S, Hammami N (2023). Intracranial dural arteriovenous fistulas: a systematic approach—diagnosis, classification, and endovascular treatment. Arab J Intervent Radiol.

[18] Zhang G, Pang M, Duan G (2025). Transarterial embolization of anterior cranial fossa dural arteriovenous fistulas as a first-line approach: A retrospective single-center study. Acta Neurochir (Wien).

[19] Lowell AE, Calgi MP, Caruso JJ, Man LM, McNeil JS (2024). Perioperative management of hemophilia patients. Curr Anesthesiol Rep.

